# Development of the Gutti Score: A Universal Clinical Assessment Framework for Patients Receiving Gabapentinoids, Opioids, Benzodiazepines, and Other Central Nervous System Depressants

**DOI:** 10.7759/cureus.109507

**Published:** 2026-05-23

**Authors:** Sai Gutti, Salah Riyadh

**Affiliations:** 1 Anesthesiology and Palliative Care, Pikeville Medical Center, Pikeville, USA; 2 Anesthesiology and Palliative Care, University of Pikeville, Pikeville, USA

**Keywords:** benzodiazepines, central nervous system depressants, chornic pain, gabapentin, interventional pain medicine, multi-modality pain management, opoids, overdose risk, pain scores, risk stratification

## Abstract

Introduction: The use of gabapentinoids, opioids, benzodiazepines, and other central nervous system (CNS) depressants has increased significantly, raising concerns about respiratory depression and overdose risk, particularly when used in combination. Existing risk assessment tools primarily focus on opioids and do not comprehensively account for multiple medication classes and patient comorbidities.

Objective: To develop and retrospectively apply a structured clinical framework (“Gutti Score”) intended to organize medication-related and patient-specific factors associated with overdose risk among patients receiving gabapentinoids, opioids, benzodiazepines, and other central nervous system depressants.

Methods: A retrospective observational pilot study was conducted using an existing patient database from 2017 to 2019. Patients prescribed gabapentinoids (gabapentin or pregabalin), opioids, benzodiazepines, or other CNS depressants were included. A structured scoring framework incorporating medication exposure and comorbid conditions was retrospectively applied using exploratory cumulative burden categories. Patients were followed for one year, and outcomes included overdose events and mortality.

Results: A total of 112 patients were included. Forty-six patients (41.1%) demonstrated lower cumulative burden profiles, while sixty-six patients (58.9%) demonstrated intermediate cumulative burden profiles. No overdose events or overdose-related deaths were observed during the follow-up period, limiting assessment of predictive performance. Patients within the intermediate cumulative burden category demonstrated a higher burden of comorbid conditions and greater medication exposure compared with those in the lower cumulative burden category.

Conclusion: These findings support the feasibility of the proposed framework; however, no conclusions regarding predictive validity or discriminatory performance can be established from the present study. Further prospective investigation involving larger and more diverse populations is required before broader clinical applicability can be determined.

## Introduction

Gabapentinoids, opioids, benzodiazepines, and other central nervous system (CNS) depressants are widely used in the management of chronic neuropathic pain, non-cancer pain, postherpetic neuralgia, seizures, and psychiatric conditions. However, their use, particularly in combination, has been associated with an increased risk of respiratory depression and overdose [1-4].

Although gabapentin is generally considered a safe medication, studies have demonstrated that its use alone or in combination with opioids can increase the risk of respiratory depression and opioid-related mortality [1,5]. Among patients receiving prescription opioids, concomitant gabapentin use has been associated with a significantly increased risk of opioid-related death [1].

The opioid epidemic continues to be a major public health concern, with opioid-related deaths exceeding those from several other major causes combined [6]. Concurrent use of benzodiazepines and other CNS depressants further amplifies the risk of overdose and mortality [3,4].

Multiple patient-related factors further increase this risk, including advanced age, chronic kidney disease, chronic obstructive pulmonary disease (COPD), liver disease, sleep apnea, psychiatric disorders, and substance use disorders [7-10].

Despite these risks, current clinical tools primarily focus on opioid-related risk and often do not provide a comprehensive approach incorporating multiple medication classes and patient-specific comorbid conditions.

The objective of this pilot study was to develop and retrospectively apply the Gutti Score, a structured clinical framework intended to provide a practical approach for organizing medication-related and patient-specific factors associated with overdose risk in patients receiving gabapentinoids, opioids, benzodiazepines, and other CNS depressants.

## Materials and methods

Study design

This study was designed as a retrospective observational pilot study using an existing patient-centered database from 2017 to 2019.

Study population

Patients prescribed gabapentinoids (gabapentin or pregabalin), opioids, benzodiazepines, or other central nervous system (CNS) depressants were included. For the purposes of this study, other CNS depressants included additional sedating medications identified within the study cohort, such as sedating antidepressants (e.g., amitriptyline, trazodone, doxepin), sedative hypnotics (e.g., zolpidem), antipsychotics (e.g., quetiapine), and similar centrally acting medications with sedative properties.

Inclusion and exclusion criteria

Patients were included if they were 18 years of age or older, were prescribed gabapentinoids (gabapentin or pregabalin), opioids, benzodiazepines, or other central nervous system (CNS) depressants, and were managed within the outpatient clinic database between 2017 and 2019. Additionally, patients were required to have available clinical follow-up data for approximately one year.

Patients were excluded if prior clinical screening identified elevated concern for opioid misuse or behavioral risk factors requiring alternative clinical management strategies.

Development of the Gutti score framework

The Gutti Score framework was developed using a literature-informed and clinically weighted approach intended to organize medication-related and patient-specific factors associated with overdose risk. Variable selection and point assignment were informed by published literature describing respiratory depression, overdose events, medication safety, and comorbidity-related risk, together with observed prescribing patterns within the retrospective study cohort. Medication exposure was quantified using standardized measures, including morphine milligram equivalents (MME) for opioids and diazepam milligram equivalents (DME) for benzodiazepines. Gabapentinoid exposure was categorized according to observed dose ranges within the study cohort. Diazepam milligram equivalents (DME) were used as a standardized measure of benzodiazepine exposure, allowing different benzodiazepine agents to be represented on a common dose scale for comparative assessment within the scoring framework. Benzodiazepine exposure categories were assigned according to standardized VA/DoD diazepam-equivalent conversion guidance summarized in the American Society of Addiction Medicine (ASAM) recommendations [11].

The framework incorporates 10 clinical variables, including medication-related factors (gabapentinoid dose, opioid dose, benzodiazepine use, and other CNS depressants) and patient-specific risk factors (age, renal function, pulmonary disease, liver disease, sleep apnea, and psychiatric or substance use disorders). Each variable was assigned a weighted score based on the relative association between the clinical factor and overdose risk as reported in existing literature, with greater weighting assigned to factors associated with increased respiratory depression, overdose events, or mortality.

Cumulative scores represented increasing medication exposure and comorbidity burden within the proposed framework. For descriptive purposes within this pilot cohort, cumulative scores were grouped into exploratory categories: lower cumulative burden (≤4), intermediate cumulative burden (5-7), and higher cumulative burden (≥8). These categories were intended solely to organize retrospective observations within the study population and were not designed or validated as predictive clinical thresholds. The detailed components and operational scoring criteria of the proposed Gutti Score framework are presented in Table 1.

**Table 1:** Gutti Score Framework and Operational Scoring Criteria The Gutti score is a structured clinical framework designed to organize medication-related and patient-specific factors associated with overdose risk among patients receiving gabapentinoids, opioids, benzodiazepines, and other central nervous system (CNS) depressants. Other CNS depressants included sedating antidepressants (e.g., amitriptyline, trazodone, doxepin), sedative hypnotics (e.g., zolpidem), antipsychotics (e.g., quetiapine), muscle relaxants, and other centrally acting sedating medications. This framework is intended to support structured clinical assessment and does not establish predictive validity or replace clinical judgment. CNS: Central Nervous System, MME: Morphine Milligram Equivalents, DME: Diazepam Milligram Equivalents, COPD: Chronic Obstructive Pulmonary Disease, CrCl: Creatinine Clearance, AHI: Apnea-Hypopnea Index, SUD: Substance Use Disorder, OUD: Opioid Use Disorder

Risk Factor	Criteria	Score
Gabapentinoids (Gabapentin/Pregabalin)	No gabapentinoid use	0
	Gabapentin ≤900 mg/day or Pregabalin ≤150 mg/day	1
	Gabapentin 901–1800 mg/day or Pregabalin 151–300 mg/day	2
	Gabapentin >1800 mg/day or Pregabalin >300 mg/day	3
Opioids (Morphine Milligram Equivalents, MME)	No opioid exposure	0
	≤15 MME/day	1
	16–30 MME/day	2
	>30 MME/day	3
Benzodiazepines (Diazepam Milligram Equivalents, DME)	No benzodiazepine use	0
	Diazepam equivalent ≤10 mg/day	1
	Diazepam equivalent 11–20 mg/day	2
	Diazepam equivalent >20 mg/day	3
Other CNS Depressants	No additional CNS depressants	0
	One concurrent sedating medication*	1
	Two or more concurrent sedating medications	2
Age	<65 years	0
	≥65 years	1
Renal Disease	Chronic kidney disease or CrCl <60 mL/min	1
Pulmonary Disease	COPD or chronic pulmonary disease	1
Liver Disease	Chronic liver disease	1
Sleep Apnea	Diagnosed obstructive sleep apnea	1
Psychiatric/Substance Use Risk	Significant psychiatric illness, suicidality, substance use disorder, or opioid use disorder	2

Rationale for dose categorization and point assignment

The scientific rationale underlying variable selection and point assignment within the Gutti Score framework is summarized in Table 2. Dose thresholds and weighted point assignments were developed using a literature-informed and clinically weighted framework intended to organize cumulative medication exposure and patient-specific risk factors associated with overdose-related outcomes. Thresholds were not selected to establish direct pharmacologic equivalence among gabapentinoids, opioids, and benzodiazepines, but rather to reflect increasing cumulative clinical burden and complexity.

**Table 2:** Scientific Rationale and Literature Basis for Variable Inclusion and Point Assignment Within the Gutti Score Framework Scientific rationale underlying variable inclusion and weighted point assignment within the Gutti Score framework. Variables were selected using a combination of observed medication exposure patterns within the retrospective study cohort, published literature describing associations with respiratory depression, overdose risk, mortality, and adverse outcomes, as well as clinical judgment derived from outpatient pain management practice. Point assignments were intended to represent exploratory cumulative clinical burden rather than direct pharmacologic equivalence among gabapentinoids, opioids, benzodiazepines, or other medication classes. Supporting evidence summarizes representative literature informing inclusion of each variable. MME: Morphine Milligram Equivalents; DME: Diazepam Milligram Equivalents; CNS: Central Nervous System; COPD: Chronic Obstructive Pulmonary Disease; SUD: Substance Use Disorder; OUD: Opioid Use Disorder.

Variable/domain	Rationale for inclusion and weighting	Supporting references
Opioid exposure, MME	Included because opioid exposure is strongly associated with respiratory depression, overdose risk, and mortality in a dose dependent manner. Published opioid overdose literature has utilized exposure categories including 1–19, 20–49, 50–99, and ≥100 MME/day, with increasing exposure associated with progressively greater overdose risk. Within this cohort, opioid exposure was generally ≤15 MME/day; therefore, higher categories within the framework were intended to represent increasing exposure burden rather than validated predictive thresholds.	1,6,12,13
Benzodiazepine exposure, DME	Included because benzodiazepines contribute to cumulative CNS and respiratory depressant burden and have been associated with adverse outcomes when combined with opioids and other sedating medications. DME was standardized using United States Department of Veterans Affairs and Department of Defense (VA/DoD) diazepam-equivalent conversion guidance summarized in American Society of Addiction Medicine (ASAM) recommendations and was used to standardize benzodiazepine exposure across agents rather than imply equivalence with MME or gabapentinoid dose.	4,11,19
Gabapentinoid exposure	Included because gabapentinoids have been associated with respiratory depression, misuse potential, altered mental status at higher doses, and increased risk when combined with opioids or in patients with renal impairment. Dose categories were informed by observed prescribing patterns within the retrospective cohort and supported by published therapeutic dosing ranges. Gabapentin thresholds near 900 mg/day and 1800 mg/day correspond to commonly utilized titration and maintenance dosing ranges, while higher categories were intended to reflect increasing medication exposure burden rather than validated predictive thresholds.	1,2,7,14
Other CNS depressants	Included because concurrent sedating medications may increase cumulative CNS depressant burden. In this cohort, these included sedating antidepressants, sedative hypnotics, antipsychotics, muscle relaxants, and similar centrally acting sedating medications.	4,5
Age ≥65 years	Included because older adults are at higher risk for medication-related sedation, respiratory depression, and adverse neurologic effects.	14,12
Renal disease	Included because impaired renal function can reduce gabapentinoid clearance and increase toxicity risk.	7,16
COPD or chronic pulmonary disease	Included because reduced pulmonary reserve may increase vulnerability to respiratory depression from opioids, gabapentinoids, and other CNS depressants.	8,12
Sleep apnea	Included because sleep-disordered breathing may increase vulnerability to opioid-related and sedative-related respiratory compromise.	17,18
Psychiatric illness, suicidality, SUD/OUD	Included because psychiatric comorbidity and substance use history may increase risk for medication misuse, unsafe combinations, and overdose-related outcomes.	2,9,19

Morphine milligram equivalent (MME) categories were informed by observed prescribing patterns within the retrospective study cohort and supplemented by published opioid overdose literature demonstrating increasing risk across daily morphine equivalent exposure categories [12,13]. Prior studies evaluating overdose risk have utilized exposure ranges including 1-19, 20-49, 50-99, and ≥100 MME/day [13]. Within this cohort, most patients received ≤15 MME/day; therefore, this threshold was selected as the lowest exposure category to reflect the predominant prescribing pattern within the study population and relatively limited opioid exposure burden.

Benzodiazepine exposure was standardized using diazepam milligram equivalents (DME). Dose equivalencies were based on the United States Department of Veterans Affairs and Department of Defense (VA/DoD) benzodiazepine equivalency values summarized in ASAM benzodiazepine tapering guidance [11]. The 10 mg diazepam-equivalent value represented one standardized equivalency unit within the VA/DoD conversion framework and served as the anchor for progressive exposure categories. The DME thresholds were selected to reflect increasing benzodiazepine exposure burden, with ≤10 DME corresponding to a low daily diazepam-equivalent dose category described in ASAM guidance, and higher categories representing progressively greater sedative burden.

Gabapentinoid categories reflected dose ranges observed within the retrospective study population and literature describing dose-related adverse neurologic effects, misuse potential, and toxicity risk in susceptible patients [2,7,14]. Point escalation was intentionally non-linear and designed to reflect progressively increasing cumulative burden rather than validated predictive risk. This approach was intended to reflect disproportionate increases in cumulative medication burden and clinical complexity at higher exposure levels.

Outcomes and follow-up

Patients were followed retrospectively for approximately one year. Outcomes of interest included overdose events, non-fatal overdose, and mortality. Patients were monitored using prescription drug monitoring programs and urine drug screening when available. Descriptive statistics were used to summarize patient demographics, medication exposure, comorbid conditions, and risk category distribution.

Statistical analysis

Descriptive statistics were used to summarize patient characteristics, risk category distribution, and medication exposure. Continuous variables were described using ranges, and categorical variables were summarized using frequencies and percentages. Due to the pilot nature of the study and absence of outcome events, no inferential statistical analysis was performed.

Ethical considerations

This retrospective study utilized de-identified patient data and was reviewed by the Institutional Review Board (IRB), which determined the study to be exempt from further IRB review due to the use of existing retrospective data. The study was conducted in accordance with institutional and ethical standards for research involving human subjects.

## Results

A total of 112 patients met the inclusion criteria. The majority of patients were over the age of 50 and predominantly Caucasian. Using exploratory cumulative burden categories within the proposed framework, 46 patients (41.1%) were classified as having lower cumulative burden (score ≤4), while 66 patients (58.9%) demonstrated intermediate cumulative burden (score 5-7). One patient with a higher cumulative burden profile (score ≥8) was excluded from the final analysis due to non-compliance and discontinuation of care.

All patients were prescribed opioids at doses ≤15 morphine milligram equivalents (MME) per day, with most receiving gabapentinoids at varying doses. A subset of patients had comorbid conditions, including chronic kidney disease and chronic obstructive pulmonary disease, which required medication dose adjustments. Patients were monitored using prescription drug monitoring programs and serial urine drug testing throughout the study period. No overdose events, non-fatal overdose incidents, or overdose-related deaths were observed during the one-year follow-up period.

Baseline clinical characteristics stratified by exploratory cumulative burden category are summarized in Table 3. Patients within the intermediate cumulative burden category demonstrated a higher prevalence of comorbid conditions, including chronic kidney disease and chronic obstructive pulmonary disease, as well as greater cumulative exposure to gabapentinoids and concurrent central nervous system depressants compared with those in the lower cumulative burden category. These findings reflect increasing clinical complexity within the study cohort and should not be interpreted as evidence of predictive validity.

**Table 3:** Baseline Characteristics by Exploratory Cumulative Burden Category Baseline clinical characteristics of patients stratified according to exploratory cumulative burden categories within the Gutti Score framework. Patients with greater cumulative burden demonstrated higher rates of comorbid conditions, polypharmacy, and concurrent CNS depressant exposure. These findings describe increasing clinical complexity within the study cohort and should not be interpreted as evidence of predictive validity. CNS: Central Nervous System, MME: Morphine Milligram Equivalents, CKD: Chronic Kidney Disease, COPD: Chronic Obstructive Pulmonary Disease.

Characteristic	Lower cumulative burden (≤4) (n = 46)	Intermediate cumulative burden (5–7) (n = 66)
Age >50 years	32 (70%)	53 (80%)
Race (Caucasian)	39 (85%)	56 (85%)
Opioid use (≤15 MME)	46 (100%)	66 (100%)
Gabapentinoid use	30 (65%)	53 (80%)
Benzodiazepine use	5 (11%)	17 (26%)
Other CNS depressants	7 (15%)	20 (30%)
Polypharmacy (≥2 CNS agents)	12 (26%)	36 (55%)
Chronic kidney disease (CKD)	2 (4%)	10 (15%)
Chronic obstructive pulmonary disease (COPD)	2 (4%)	8 (12%)
Liver disease	1 (2%)	5 (8%)
Sleep apnea	1 (2%)	7 (11%)
Psychiatric / substance use disorders	9 (20%)	26 (39%)

## Discussion

This study presents a structured clinical framework intended to organize medication-related and patient-specific factors associated with overdose risk across multiple medication classes, including gabapentinoids, opioids, benzodiazepines, and additional central nervous system (CNS) depressants. In this study, additional CNS depressants included sedating antidepressants, sedative hypnotics, antipsychotics, and other centrally acting medications with sedative properties identified within the study cohort.

Unlike existing approaches that focus primarily on opioid use, this framework incorporates multiple pharmacologic exposures and comorbid conditions, providing a broader multidimensional assessment of factors associated with overdose risk. The integration of these variables into a structured multidimensional framework distinguishes this approach from existing models and highlights its potential utility in organizing medication-related and patient-specific risk factors during clinical assessment. To our knowledge, few published approaches have attempted to integrate gabapentinoids, opioids, benzodiazepines, and comorbid conditions into a unified overdose risk assessment framework.

The absence of overdose events in this cohort may reflect cautious prescribing practices, including the use of opioid doses ≤15 MME/day and close clinical monitoring. Prior studies have demonstrated that gabapentinoids, particularly when combined with opioids, are associated with an increased risk of opioid-related death and respiratory depression [1,3]. Additionally, concurrent use of benzodiazepines, z-drugs, and gabapentinoids in patients receiving opioid therapy has been associated with increased mortality risk [4]. Clinical prescribing guidelines further emphasize the importance of cautious opioid dosing and avoidance of high-risk combinations [6].

Gabapentinoids have also been increasingly recognized for their misuse and abuse potential, particularly among individuals with substance use disorders [2,9]. Epidemiologic data have demonstrated the presence of gabapentin in postmortem toxicology analyses of overdose cases, further supporting its role in overdose risk [10].

Higher doses of gabapentin have been associated with adverse neurologic effects, particularly in older adults [14]. Gabapentinoid misuse has also been linked to opioid addiction, further compounding overdose risk in vulnerable populations [15]. Additionally, gabapentin toxicity has been well described in patients with impaired renal function, highlighting the importance of dose adjustment in this population [16]. Chronic opioid use has also been associated with central sleep apnea and respiratory instability [17], and gabapentinoids have been shown to increase the apnea-hypopnea index in susceptible individuals [18]. Management of benzodiazepine misuse and dependence remains an important clinical consideration when addressing polypharmacy and overdose risk [19]. Additionally, pharmacologic transitions between gabapentinoids, such as substitution of gabapentin with pregabalin, may influence clinical management strategies in select patient populations [20].

Patients demonstrating greater cumulative burden within the proposed framework exhibited higher rates of comorbid conditions and increased medication exposure compared with those demonstrating lower cumulative burden. These findings support the internal consistency of the framework in identifying increasing clinical complexity, even in the absence of observed overdose events. Mechanistic insights into gabapentinoid pharmacology suggest that these agents may potentiate central nervous system and respiratory depression, particularly when combined with other sedating medications [5].

Importantly, this study was not designed to evaluate predictive accuracy but rather to assess the feasibility and clinical applicability of the scoring framework in a real-world clinical setting. The relatively low cumulative burden and closely monitored nature of this study population, together with the absence of overdose events, limit conclusions regarding discriminatory ability or predictive validity.

Patients with a history of substance use disorder (SUD), opioid use disorder (OUD), prior non-fatal overdose, or significant psychiatric comorbidities represent a higher-risk population for overdose. Concurrent use of benzodiazepines and opioids has been associated with increased risk of adverse outcomes and requires careful monitoring and management [4,19].

Comorbid conditions such as chronic kidney disease and pulmonary disease further increase the risk of adverse outcomes in patients receiving CNS depressants. Opioid use has been associated with adverse respiratory outcomes in patients with chronic obstructive pulmonary disease [8], and renal impairment contributes to the accumulation and toxicity of gabapentinoids [16].

The proposed framework may provide a structured approach for organizing medication exposure and patient-specific characteristics associated with overdose risk during routine clinical assessment. While potentially useful for identifying modifiable clinical factors such as medication burden and concurrent sedating therapies, the present findings should be interpreted as exploratory and feasibility-based rather than as evidence of validated clinical prediction.

However, several limitations must be acknowledged. The study was conducted at a single center with a relatively homogeneous and closely monitored patient population, which may limit generalizability. Importantly, no overdose events or overdose-related adverse outcomes occurred during follow-up, limiting assessment of discriminatory ability and preventing conclusions regarding predictive validity. Additionally, the limited representation of higher cumulative burden patients and the retrospective study design restrict broader interpretation and preclude causal inference.

Further prospective studies involving larger and more diverse patient populations, including higher cumulative burden groups and varying medication regimens, are necessary to further evaluate the feasibility, reproducibility, and potential clinical applicability of the proposed framework.

## Conclusions

This retrospective observational pilot study describes the development and retrospective application of a structured clinical framework (Gutti Score) intended to organize medication-related factors and patient-specific comorbid conditions associated with overdose risk among patients receiving gabapentinoids, opioids, benzodiazepines, and additional central nervous system (CNS) depressants. Rather than serving as a validated predictive model, the proposed framework was developed to provide a structured approach for assessing cumulative medication exposure and clinical complexity in patients exposed to multiple CNS depressants.

Within this cohort, most patients demonstrated lower or intermediate cumulative burden profiles, and no overdose events or overdose-related deaths were observed during follow-up. These findings likely reflect a relatively low cumulative burden, a closely monitored population with cautious prescribing practices. While the present study supports the feasibility of implementing this multidimensional assessment approach, the absence of adverse outcome events limits conclusions regarding discriminatory ability or predictive validity. Further prospective investigation involving larger and more diverse populations is required before broader clinical applicability can be determined.

## References

[1] Gomes T, Juurlink DN, Antoniou T (2017). Gabapentin, opioids, and the risk of opioid-related death: a population-based nested case-control study. PLoS Med.

[2] Smith RV, Havens JR, Walsh SL (2016). Gabapentin misuse, abuse and diversion: a systematic review. Addiction.

[3] Savelloni J, Gunter H, Lee KC (2017). Risk of respiratory depression with opioids and concomitant gabapentinoids. J Pain Res.

[4] Macleod J, Steer C, Tilling K (2019). Prescription of benzodiazepines, z-drugs, and gabapentinoids and mortality risk in people receiving opioid agonist treatment: Observational study based on the UK Clinical Practice Research Datalink and Office for National Statistics death records. PLoS Med.

[5] McAnally H, Bonnet U, Kaye AD (2020). Gabapentinoid benefit and risk stratification: mechanisms over myth. Pain Ther.

[6] Dowell D, Haegerich TM, Chou R (2016). CDC guideline for prescribing opioids for chronic pain--United States, 2016. JAMA.

[7] Raouf M, Atkinson TJ, Crumb MW, Fudin J (2017). Rational dosing of gabapentin and pregabalin in chronic kidney disease. J Pain Res.

[8] Vozoris NT, Wang X, Fischer HD (2016). Incident opioid drug use and adverse respiratory outcomes among older adults with COPD. Eur Respir J.

[9] Hägg S, Jönsson AK, Ahlner J (2020). Current evidence on abuse and misuse of gabapentinoids. Drug Saf.

[10] Slavova S, Miller A, Bunn TL (2018). Prevalence of gabapentin in drug overdose postmortem toxicology testing results. Drug Alcohol Depend.

[11] (2026). Joint Clinical Practice Guideline on Benzodiazepine Tapering: Benzodiazepine dose equivalents. https://downloads.asam.org/sitefinity-production-blobs/docs/default-source/guidelines/benzodiazepine-tapering-2025/benzodiazepine-dose-equivalents.pdf.

[12] Dowell D, Ragan KR, Jones CM (2022). CDC clinical practice guideline for prescribing opioids for pain - United States, 2022. MMWR Recomm Rep.

[13] Liang Y, Turner BJ (2015). Assessing risk for drug overdose in a national cohort: role for both daily and total opioid dose?. J Pain.

[14] Fleet JL, Dixon SN, Kuwornu PJ (2018). Gabapentin dose and the 30-day risk of altered mental status in older adults: a retrospective population-based study. PLoS One.

[15] Bastiaens L, Galus J, Mazur C (2016). Abuse of gabapentin is associated with opioid addiction. Psychiatr Q.

[16] Zand L, McKian KP, Qian Q (2010). Gabapentin toxicity in patients with chronic kidney disease: a preventable cause of morbidity. Am J Med.

[17] Correa D, Farney RJ, Chung F (2015). Chronic opioid use and central sleep apnea: a review of the prevalence, mechanisms, and perioperative considerations. Anesth Analg.

[18] Piovezan RD, Kase C, Moizinho R (2017). Gabapentin acutely increases the apnea-hypopnea index in older men: data from a randomized, double-blind, placebo-controlled study. J Sleep Res.

[19] Brett J, Murnion B (2015). Management of benzodiazepine misuse and dependence. Aust Prescr.

[20] Toth C (2010). Substitution of gabapentin therapy with pregabalin therapy in neuropathic pain due to peripheral neuropathy. Pain Med.

